# Modulation of the immune response in HTLV-1-infections by SM29 antigen in vitro is dependent of IL-10, TGF-β and CTLA-4

**DOI:** 10.1186/1742-4690-11-S1-P68

**Published:** 2014-01-07

**Authors:** Luciane Mota Lima, Silvane Braga Santos, Ricardo Riccio Oliveira, Luciana Santos Cardoso, Sergio Costa Oliveira, Alfredo Miranda Góes, Alex Loukas, Edgar M De Carvalho, Maria Ilma Araújo

**Affiliations:** 1 Serviço de Imunologia, Hospital Universitário Professor Edgard Santos, Universidade Federal da Bahia, Salvador-BA, Brazil; 2 Instituto de Ciências Biológicas, Departamento de Bioquímica e Imunologia, Universidade Federal de Minas Gerais, Belo Horizonte-MG, Brazil; 3 Division of Infectious Diseases, Queensland Institute of Medical Research, Brisbane, Queensland, Australia

## 

The HTLV-1 is the causal agent of HTLV-1-associated Myelopathy/Tropical Spastic Paraparesis (HAM/TSP). The immune response in HTLV-1 infection is polarized to the Th1-type, and previous data from our group had demonstrated that the use of *Schistosoma* antigens to cell cultures in HTLV-1 infection resulted in down-modulation of Th1/inflammatory response. The aim of this study was to evaluate whether the modulation of inflammatory response by *S. mansoni* antigens depends upon the presence of IL-10, TGF-β and CTLA-4. The antibodies anti-IL-10, anti-TGF-β and anti-CTLA-4 with or without the *S. mansoni* antigen Sm29 were added to the PBMC cultures of HTLV-1- infected individuals and the levels of cytokines in the supernatants were measure using ELISA sandwich method. Compared to the levels of cytokine in non stimulated cultures (1.073 pg/ml, median values), the levels of IFN-gproduction (552 pg/ml, 1.002 pg/ml and 548 pg/ml, respectively; p<0.05). On the other hand, the levels of IL-10 were increased by the presence of Sm29 (it passed from 26 pg/ml to102 pg/ml; p=0.02), however when anti-TGF-β and anti-CTLA-4 were added to the cultures there was a reduction in the levels of IL-10 (19 pg/ml and 23 pg/ml, respectively; p<0.05). The regulation of IFN-y production by Sm29 antigen in HTLV-1-infected individuals is dependent on IL-10, TGF-β and CTLA-4. Also the levels of IL-10 were affected by the neutralization of TGF-β and CTLA-4.

## Financial support

CNPQ (Universal 479417/2008 3), NIH (R01AI079238A).

